# Interaction Between *Bacillus subtilis* and *Trichoderma citrinoviride*: Synergistic Inhibition of *Aspergillus flavus* Growth and Aflatoxin B_1_ Production

**DOI:** 10.3390/toxins18030119

**Published:** 2026-02-26

**Authors:** Guidong Li, Xianfeng Ren, Changying Guo, Baocheng Xu, Xinjing Dou

**Affiliations:** 1 College of Food and Bioengineering, Henan University of Science and Technology, Luoyang 471003, China; 2 Institute of Agricultural Quality Standards and Testing Technology, Shandong Academy of Agricultural Sciences, Jinan 250100, China; 3 Shandong Provincial Key Laboratory of Test Technology on Food Quality and Safety, Jinan 250100, China

**Keywords:** biocontrol, *Bacillus subtilis*, *Trichoderma citrinoviride*, *Aspergillus flavus*, culture filtrate

## Abstract

*Aspergillus flavus* (*A. flavus*) and its potent carcinogenic metabolite, aflatoxin B_1_ (AFB_1_), pose severe threats to food safety and public health. This study explored the biocontrol potential of *Bacillus* and *Trichoderma* strains and their interactions. Through dual-culture assays, *Bacillus subtilis* Bs92 and *Trichoderma citrinoviride* GC-T20 were identified as the most inhibitory among 15 isolates each, with their culture filtrates inhibiting *A. flavus* growth by 48.61% and 74%, respectively. Investigation of the inter-genus interaction revealed strong mutual inhibition: *Trichoderma* filtrate suppressed *Bacillus* growth by >90%, while *Bacillus* filtrate inhibited *Trichoderma* mycelial growth by 40–90%. To circumvent this antagonism and enhance efficacy, the culture filtrates of Bs92 and GC-T20 were combined. The combined treatment demonstrated superior performance, inhibiting *A. flavus* radial growth by 69.59% and AFB_1_ production by 98.69%, significantly outperforming individual applications. These results indicate that the antifungal and anti-aflatoxigenic metabolites produced by Bs92 and GC-T20 are complementary. Their combined use presents a promising synergistic strategy for the biocontrol of *A. flavus* and AFB_1_ contamination in food and agricultural products.

## 1. Introduction

*Aspergillus flavus (A. flavus*) is a naturally occurring pathogenic microorganism that causes aflatoxin contamination, leading to food waste and economic losses [1]. It commonly infects a variety of food and agricultural products, including crops, grains, and animal feed [2]. Upon contamination, *A. flavus* rapidly multiplies by producing abundant mycelia and conidia [3]. During growth, the fungus consumes nutrients from the host plants, thereby impairing their normal development [4]. Moreover, *A. flavus* produces several types of aflatoxins, which pose serious risks to food safety and human health [5]. Among these, aflatoxin B_1_ (AFB_1_)—known for its strong hepatotoxicity, teratogenicity, and developmental toxicity—has been classified as a Group 1 carcinogen by the International Agency for Research on Cancer (IARC) [6]. Currently, AFB_1_ contamination remains a severe problem worldwide, calling for urgent control measures [7].

Common strategies for controlling fungi and mycotoxins include physical, chemical, and biological approaches [8]. The contamination of AFB_1_ can be effectively mitigated by inhibiting the growth of *A. flavus* [9]. Among these, biological methods offer advantages such as low cost, high specificity, environmental safety, and minimal impact on food quality [10]. These methods utilize various microorganisms—including bacteria and fungi—that can suppress the growth of *A. flavus* and the production of AFB_1_ [11]. Among bacterial biocontrol agents, *Bacillus* species are particularly promising due to their ability to produce a wide array of antifungal compounds, notably lipopeptides [12,13]. For instance, Yuan et al. isolated a strain of *B. subtilis* that demonstrated a 64.27% inhibition rate against *A. flavus*, and its fermentation supernatant was shown to damage the fungal mycelia [14]. Further study has revealed that lipopeptides in the supernatant, such as surfactant and fengycin, can inhibit both the growth of *A. flavus* and AFB_1_ production by targeting the cell walls and membranes of *A. flavus*, thereby interfering with mycelial growth and spore germination [15]. Another species, *Bacillus velezensis* (*B. velezensis*), a recently identified member of the genus, produces several antibiotics and exhibits antifungal activity [16,17]. In parallel, beneficial fungi such as *Trichoderma* species are widely distributed in soils and plant root systems and have long been recognized for their efficacy against plant pathogens [18,19]. They produce antifungal substances to counteract *A. flavus* contamination [20]. Moreover, Ren et al. investigated the inhibitory effect of 20 *Trichoderma* strains on AFB_1_ production by *A. flavus* and concluded that this suppression was not associated with the synthesis of AFB_1_ or related gene regulation, but might result from enzymatic degradation [21]. Further studies have shown that *Trichoderma citrinoviride* (*T. citrinoviride*) produces antifungal surfactants when cultured with inducers [22], while *Trichoderma asperellum* (*T. asperellum*) secretes both diffusible metabolites and volatile compounds that inhibit *A. flavus* growth [20].

It is evident that both *Bacillus* and *Trichoderma* demonstrate significant biocontrol potential against *A. flavus*, and their application in this context has been extensively studied and widely implemented [23]. With the continuous advancement of microbiomics, the construction of synthetic microbial consortia—combining different microorganisms or their metabolites—has attracted growing research interest. Moreover, since the mechanisms through which *Bacillus* and *Trichoderma* inhibit *A. flavus* are distinct yet complementary, their combined use represents a highly promising strategy. For instance, Suresh et al. used cell-free extracts from fungi and bacteria to degrade AFB_1_ and found that they produced enzymes with different functionalities, leading to varying degradation rates [24]. In another study, the simultaneous inoculation of *Bacillus* and *Trichoderma* into soil was shown to enhance soybean plant height and yield [25]. Santoyo et al. also proposed using *Bacillus* and *Trichoderma* to promote plant growth in saline soils [26]. However, while existing research has largely focused on validating the combined efficacy of *Bacillus* and *Trichoderma*, studies on the interactions between them remain limited. In particular, whether antagonistic effects occur when they are applied together—and whether such interactions could impair their respective abilities to inhibit *A. flavus*—requires further investigation.

Therefore, this study aims to explore the interaction between *Bacillus* and *Trichoderma*, building on the understanding of their individual inhibitory mechanisms against *A. flavus*. The ultimate goal is to develop a more effective combined strategy to suppress both *A. flavus* growth and AFB_1_ production. Based on culture filtrate assays and dual-culture antagonism tests, we selected two *Bacillus* strains and five *Trichoderma* strains exhibiting strong inhibitory activity against *A. flavus* from a total of 15 *Bacillus* and 15 *Trichoderma* isolates. We then conducted interspecific confrontation assays between *Bacillus* and *Trichoderma*, and further evaluated the effects of *Bacillus* culture filtrates on *Trichoderma* growth, as well as the influence of *Trichoderma* culture filtrates on *Bacillus* growth. Building on these results, we evaluated the combined use of their culture filtrates to inhibit *A. flavus* growth and AFB_1_ production. This study provides a theoretical foundation for constructing synthetic microbial consortia and offers practical insights into the integrated biocontrol of *A. flavus* using *Trichoderma* and *Bacillus*.

## 2. Results

### 2.1. Inhibitory Effects of the Bacillus Strains on A. flavus

**Figure 1:**
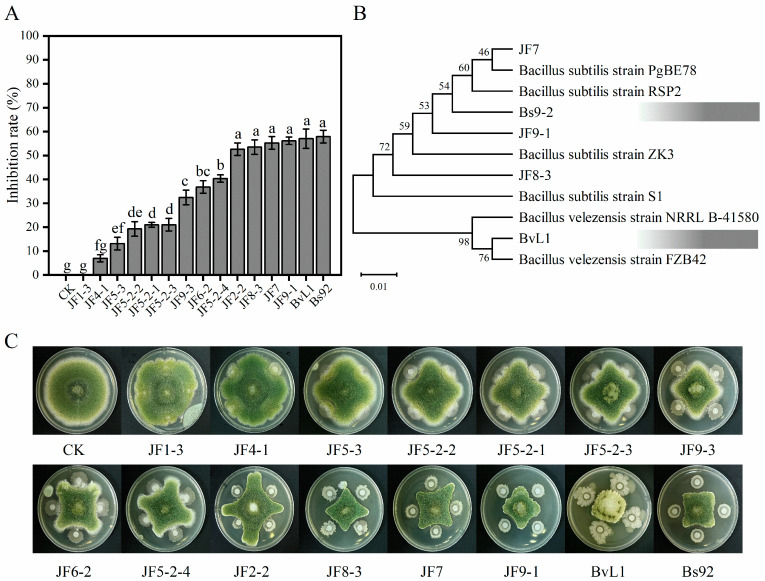
Inhibitory effects of *Bacillus* strains on *A. flavus*. (**A**) Inhibition rates of different *Bacillus* strains against *A. flavus*. (**B**) Phylogenetic trees of five *Bacillus* strains with strong antifungal ability. (**C**) Representative images of antagonistic interactions between *Bacillus* strains and *A. flavus* after 7 days of cultivation. Data in (**A**) are expressed as mean ± SD (*n* = 3). Bars labeled with different letters indicate statistically significant differences (*p* < 0.05, Tukey–Kramer test).

To identify bacteria capable of inhibiting *A. flavus*, 15 strains were isolated from the rhizosphere soil of peanut, corn, and soybean plants. The inhibitory effects of these strains against *A. flavus* are shown in Figure 1A. Among them, strains JF1-3 and JF2-2 showed no inhibitory activity. In contrast, five strains—JF8-3, JF7, JF9-1, BvL1, and Bs92—exhibited the strongest inhibition, with inhibition rates of 53.51%, 55.26%, 56.14%, 57.02%, and 57.89%, respectively. The remaining strains also showed varying degrees of inhibition. Based on these results, the five strains with the strongest inhibitory activity were selected for phylogenetic identification. According to the phylogenetic tree (Figure 1B), all five strains belonged to the genus *Bacillus*. Among them, JF8-3, JF7, JF9-1 and Bs92 were identified as *B. subtilis*, while BvL1 was identified as *B. velezensis*. As shown in Figure 1C, the inhibition zones exhibited distinct morphological patterns. In most cases, *A. flavus* grew in a cross-like shape, as fungal growth was arrested in the inhibited regions but continued in uninhibited areas. Strains with strong and broad-spectrum inhibitory effects, such as JF9-1, BvL1, and Bs92, induced a more squared growth pattern of *A. flavus*. Additionally, inhibition was generally observed only in areas directly adjacent to the *Bacillus* colonies. However, a few highly inhibitory strains produced clear zones of inhibition even without direct contact, suggesting the secretion of diffusible antifungal compounds. These observations indicate that different strains of the same *Bacillus* species may employ distinct mechanisms to inhibit *A. flavus*.

### 2.2. Inhibitory Effects of the Bacillus Strains’ Culture Filtrates on A. flavus

**Figure 2:**
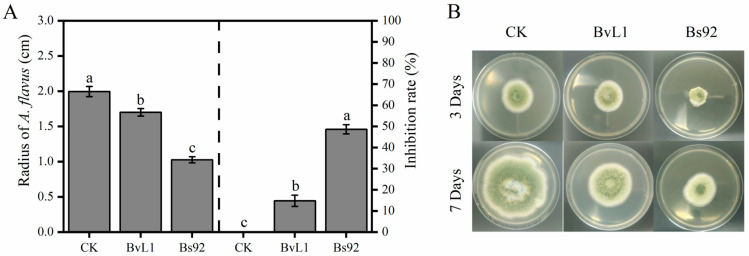
Inhibitory effects of *Bacillus* strains’ culture filtrates on *A. flavus*. (**A**) Inhibition rates of *A. flavus* growth after 3 days of exposure to culture filtrates from different *Bacillus* strains. Dates to the left of the dashed line represent the effect of the *Bacillus* strains’ culture filtrates on the growth radius of *A. flavus*, while those to the right show its inhibition rate on *A. flavus*. (**B**) Representative images showing *A. flavus* growth in the presence of *Bacillus* culture filtrates. Data represent mean ± SD (*n* = 3). Bars labeled with different letters indicate statistically significant differences (*p* < 0.05, Tukey–Kramer test).

To investigate the inhibitory effects of different *Bacillus* strains’ culture filtrates on *A. flavus*, two strains—*B. velezensis* (BvL1) and *B. subtilis* (Bs92)—were selected. As shown in Figure 2A, after 3 days of incubation, the radial growth of *A. flavus* in the control (CK) group reached 1.99 cm, whereas in treatments supplemented with culture filtrates from BvL1 and Bs92, growth was reduced to 1.64 cm and 1.03 cm, respectively. The corresponding inhibition rates were 15.79% for BvL1 and 48.61% for Bs92. After 7 days of cultivation (Figure 2B), visual assessment confirmed the inhibitory effect of both bacterial filtrates on *A. flavus* growth, with Bs92 culture filtrate exhibiting stronger suppression than that of BvL1.

### 2.3. Inhibitory Effects of the Trichoderma Strains’ Culture Filtrates on A. flavus

**Figure 3:**
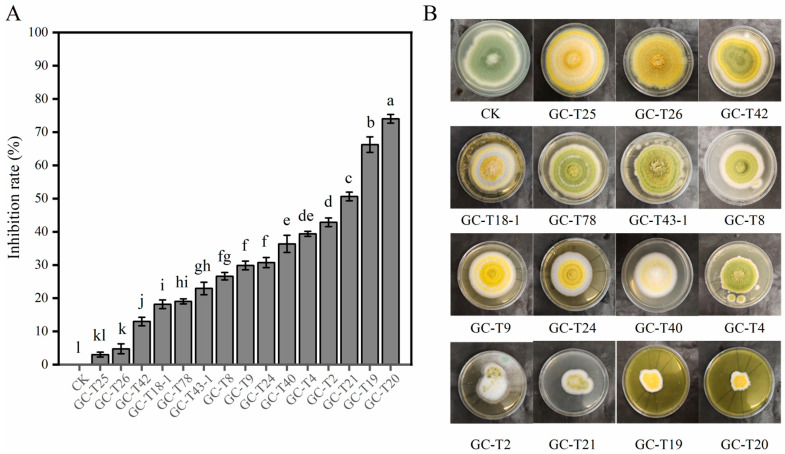
Inhibitory effects of the *Trichoderma* strains’ culture filtrates on *A. flavus*. (**A**) Inhibition rates of *A. flavus* growth by culture filtrates from different *Trichoderma* strains. (**B**) Representative images of *A. flavus* growth on PDA plates supplemented with *Trichoderma* culture filtrates after 7 days of cultivation. Data in (**A**) are expressed as mean ± SD (*n* = 3). Bars labeled with different letters indicate statistically significant differences (*p* < 0.05, Tukey–Kramer test).

The inhibitory effects of culture filtrates from the 15 *Trichoderma* strains against *A. flavus* are shown in Figure 3A. After 7 days of cultivation, differences in fungal growth were observed across treatments, with most culture filtrates suppressing the development of *A. flavus*. Notably, *T. citrinoviride* strains GC-T20 and GC-T19 exhibited the strongest inhibition, with rates of 74.03% and 66.23%, respectively. Other highly inhibitory strains included *T. citrinoviride* GC-T21 and *T. asperellum* GC-T2 and GC-T4, all of which have been described in the prior study [21]. Visual inspection further confirmed the inhibition of *A. flavus* growth. Compared to the control group, the smallest growth radii were observed in plates treated with culture filtrates from GC-T19, GC-T20, and GC-T21 (Figure 3B).

### 2.4. Plate Confrontation Experiment Between Bacillus Strains and Trichoderma Strains

**Figure 4:**
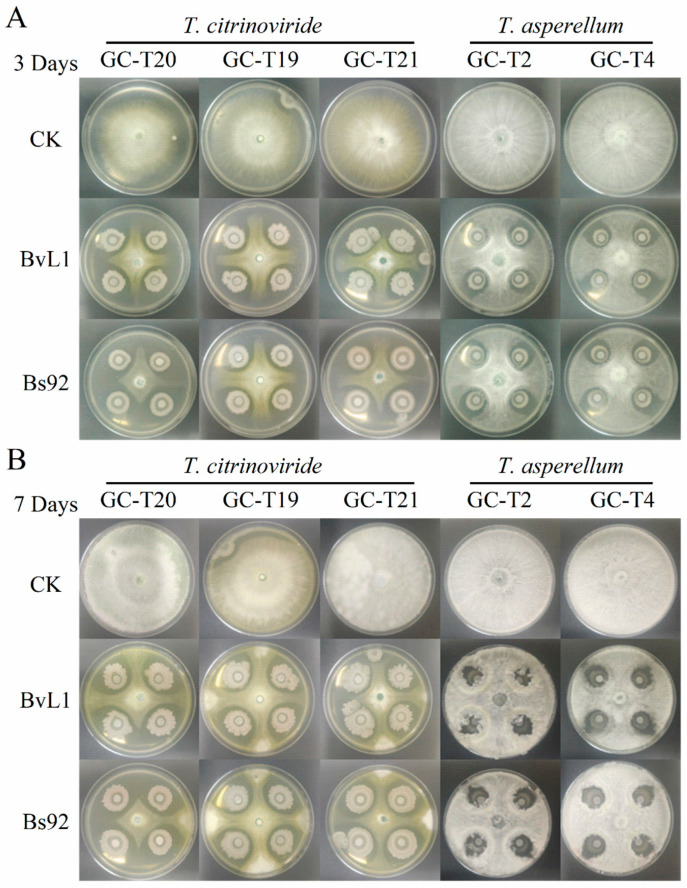
Plate confrontation experiments between *Bacillus* and *Trichoderma* strains. (**A**) Representative images of dual-culture interactions after 3 days of co-cultivation. (**B**) Representative images of antagonistic interactions after 7 days of co-cultivation.

To investigate the interactions between *Bacillus* species and *Trichoderma* species, a plate confrontation assay was performed using selected strains. As shown in Figure 4A, after 3 days of co-cultivation, *Trichoderma* strains exposed to *Bacillus* culture filtrate showed significant growth inhibition, forming a cross-shaped colony morphology. The inhibitory effects of different *Bacillus* strains against the same *Trichoderma* species were not significantly different. In contrast, the same *Bacillus* strain exhibited varying inhibition levels against different *Trichoderma* species. On the other hand, *Trichoderma* demonstrated limited inhibitory activity against *Bacillus*, which may be due to the slower growth rate of the bacteria. After 7 days of co-culture (Figure 4B), *T. citrinoviride* developed a more distinct inhibition zone when paired with *Bacillus*. These results suggest that *Bacillus* and *T. citrinoviride* mutually inhibit each other, which may affect their combined efficacy against *A. flavus*. In comparison, the antagonism between *Bacillus* and *T. asperellum* showed no clear competitive exclusion. Given that *T. asperellum* exhibits relatively low inhibitory activity against *A. flavus*, combining *Bacillus* culture filtrate with that of the more potent *T. citrinoviride* may represent a feasible strategy for constructing an effective synthetic microbial consortium against *A. flavus*.

### 2.5. Inhibitory Effects of the Trichoderma Strains’ Culture Filtrates on Bacillus Strains

**Figure 5:**
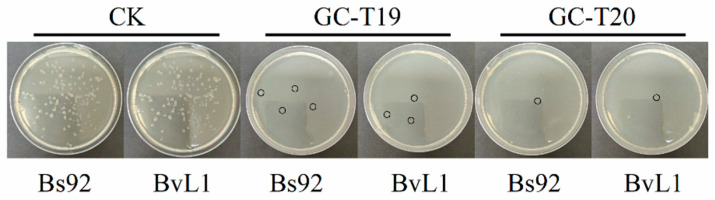
Inhibitory activity of *Trichoderma* culture filtrates against various *Bacillus* strains. Black circles correspond to *Bacillus* strains (representing visibly grown *Bacillus* strains on the culture medium). Representative photographs of colonies grown on LB agar plates from the 10^−6^ dilution.

Two *Trichoderma* strains (GC-T19 and GC-T20) with the strongest inhibitory effect on *A. flavus* were selected to evaluate the inhibitory activity of their culture filtrates against *Bacillus* in a liquid confrontation assay. After seven days of incubation, the optical density at 600 nm (OD_600_) of the different liquid cultures was measured (Table 1). The OD_600_ values of *Bacillus* strains in the control (CK) group were significantly higher than those in treatments supplemented with *Trichoderma* culture filtrate. Moreover, there was no significant difference in OD_600_ between the two *Trichoderma* filtrate treatments, both of which strongly inhibited the growth of the two *Bacillus* strains. These results indicate that the *Trichoderma* culture filtrates contain substances that markedly suppress *Bacillus* growth. As determined from the 10^−6^ dilution plates (Figure 5), control counts of Bs92 and BvL1 were 6.6 × 10^7^ CFU/mL and 5.5 × 10^7^ CFU/mL, respectively. Treatment with *Trichoderma* filtrates reduced these counts to 1–5 × 10^6^ CFU/mL.

### 2.6. Inhibitory Effects of the Bacillus Strains’ Culture Filtrates on Trichoderma Strains

**Figure 6:**
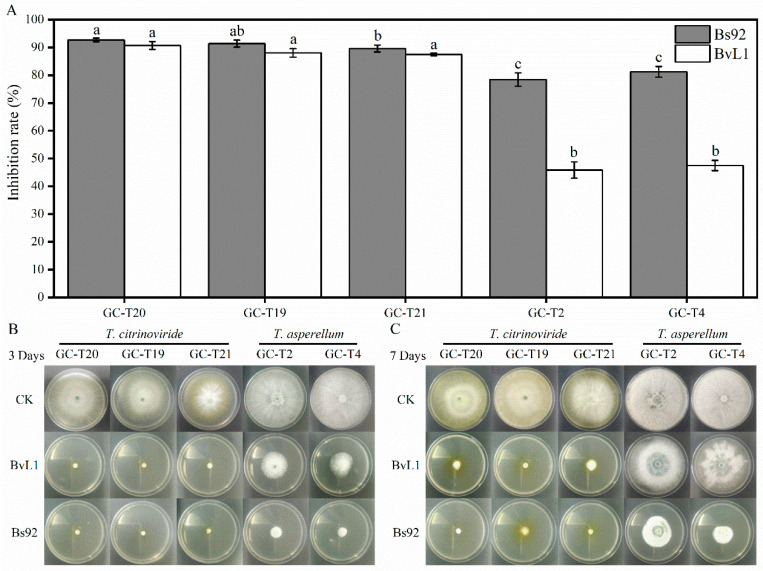
Effects of *Bacillus* strains’ culture filtrate on *Trichoderma* growth. (**A**) Inhibition rates of *Trichoderma* strains exposed to culture filtrates from different *Bacillus* strains. (**B**) Representative images of *Trichoderma* growth after 3-day exposure to *Bacillus* culture filtrates. (**C**) Representative images of *Trichoderma* growth after 7-day exposure to *Bacillus* culture filtrates. Data represent mean ± SD (*n* = 3). Bars labeled with different letters indicate statistically significant differences (*p* < 0.05, Tukey–Kramer test).

The inhibitory effects of *Bacillus* culture filtrates on *Trichoderma* strains were also evaluated. As shown in Figure 6A, culture filtrates from both Bs92 and BvL1 strongly inhibited *T. citrinoviride*, with no significant difference between them and inhibition rates exceeding 85%. In contrast, their effects on *T. asperellum* strains differed markedly: Bs92 showed inhibition rates of 78.41% against GC-T2 and 81.21% against GC-T4, while BvL1 exhibited lower inhibition rates of 45.85% and 47.48%, respectively. After 3 days of cultivation (Figure 6B), *Trichoderma* in the control group had grown to the edge of the PDA medium, whereas growth was significantly suppressed in plates supplemented with *Bacillus* culture filtrates. By day 7 (Figure 6C), the inhibitory effect became more pronounced, particularly against *T. citrinoviride*—the species previously identified as having the strongest antifungal activity against *A. flavus*. Notably, the culture filtrates of Bs92 (*B. subtilis*) and BvL1 (*B. velezensis*) differed significantly in their ability to inhibit *T. asperellum*, likely reflecting differences in their antifungal metabolite profiles. Among the two, Bs92 culture filtrate exhibited the strongest overall inhibitory activity against *Trichoderma*.

### 2.7. Inhibitory Effects of the Bacillus and Trichoderma Strain Culture Filtrates on A. flavus

**Figure 7:**
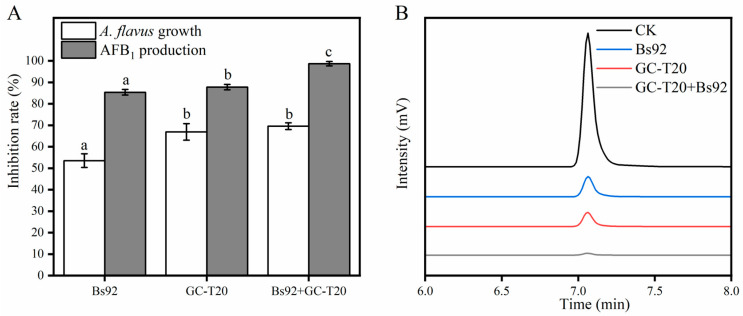
Inhibitory effects of individual and combined culture filtrates on *A. flavus*. (**A**) Inhibition rates of fungal growth and AFB_1_ production by different culture filtrates. (**B**) Representative chromatograms showing AFB_1_ levels in different treatment groups. Data represent mean ± SD (*n* = 3). Bars labeled with different letters indicate statistically significant differences (*p* < 0.05, Tukey–Kramer test).

The inhibitory effect of the combined culture filtrate of *B. subtilis* (Bs92) and *T. citrinoviride* (GC-T20) on the growth of *A. flavus* and the production of AFB_1_ was shown in Figure 7A. When applied individually, the culture filtrates of Bs92 and GC-T20 inhibited the growth of *A. flavus* by 53.53% and 66.91%, respectively. Notably, the combined filtrate of Bs92 and GC-T20 did not significantly enhance fungal growth inhibition compared to GC-T20 alone, achieving an inhibition rate of 69.59%. This suggests that in the combined treatment, the antifungal activity may be primarily attributable to the GC-T20 filtrate. Chromatographic analysis of AFB_1_ (Figure 7B) showed that the AFB_1_ concentration in the control group reached 102.82 ± 0.03 μg/mL after 7 days of incubation. In treatments with Bs92 or GC-T20 filtrates alone, AFB_1_ levels were reduced to 12.54 ± 0.01 μg/mL and 15.04 ± 0.00 μg/mL, corresponding to inhibition rates of 87.80% and 85.37%, respectively. In contrast, when both filtrates were applied together, inhibition of AFB_1_ production was markedly enhanced, with an inhibition rate of 98.69% and a final AFB_1_ concentration of only 1.35 μg/mL. In summary, the application of the combined culture filtrate of Bs92 and GC-T20 resulted in a trend toward enhanced inhibition of both *A. flavus* growth and AFB_1_ production compared to individual applications. This trend was most evident in the reduction in AFB_1_ levels, indicating a potential additive benefit of the combination for targeting aflatoxin biosynthesis.

## 3. Discussion

Numerous studies have isolated microorganisms from natural environments that exhibit inhibitory activity against fungi, particularly the highly toxigenic *A. flavus*, including strains of *Bacillus* and *Trichoderma* [14,27]. *Bacillus* species, generally recognized as safe (GRAS), have been widely used to control contamination by various fungi. For example, Taghavi et al. reported that *Bacillus subtilis*, *Bacillus licheniformis*, *Bacillus axarquiensis*, and other *Bacillus* spp. isolated from soil samples demonstrated strong antifungal activity against multiple fungal species, highlighting the potential of this genus as a promising biocontrol agent [28]. Similarly, Ma et al. isolated three strains from maize, soybean, and rice seeds that effectively inhibited *A. flavus*, which were identified as *B. velezensis* and *B. amyloliquefaciens* [29]. In the present study, two *Bacillus* strains—*B. subtilis* and *B. velezensis*—were isolated from plant rhizosphere soil and shown to inhibit *A. flavus* by nearly 60%. Research has shown that *Bacillus* species produce various antifungal compounds, especially lipopeptides, which suppress fungal growth and mycotoxin production [13,30]. For instance, Zhao et al. indicated that secondary metabolites of *B. subtilis*, particularly lipopeptides such as bacillomycin D and mycosubtilin, exhibit broad-spectrum antifungal activity and show the most significant inhibition against *A. flavus* [31]. Meanwhile, Li et al. found that *B. velezensis* primarily produces fengycin and iturins, which inhibit both the growth of *A. flavus* and aflatoxin biosynthesis [32]. Therefore, the *Bacillus* strains screened in this study are likely to inhibit the growth of *A. flavus* and the synthesis of AFB_1_ through the production of lipopeptides. Among them, the culture filtrate of *Bacillus subtilis* Bs92 exhibited the strongest inhibitory activity against *A. flavus*. This may be attributed to the higher potency of the lipopeptides it produces, which could make it more suitable for practical applications.

Based on previous findings regarding the inhibitory activity of *Trichoderma* against *A. flavus*, we further evaluated the antifungal effect of their culture filtrates. The inhibition rates of different *Trichoderma* culture filtrates on the growth of *A. flavus* varied significantly, among which *T. citrinoviride* (GC-T19, GC-T20 and GC-T21) exhibited the strongest inhibitory effect. Current studies indicate that *Trichoderma* can mitigate fungal contamination and mycotoxin production through diverse mechanisms [33]. These mechanisms are often species-specific. For instance, *T. harzianum* acts via mycoparasitism, whereas *A. flavus* produces antifungal enzymes and other chemical agents to suppress *A. flavus* [23]. In addition, Sawai et al. reported that the culture filtrate of *T. asperelloides* inhibits mycelial growth and spore germination in two toxigenic fungal strains by directly disrupting ergosterol synthesis [34]. In this study, the inhibition of *A. flavus* by the selected *Trichoderma* strains is likely mediated by certain substances present in their culture filtrates. Several reports have confirmed that *Trichoderma* secretes extracellular enzymes that impair mycelial growth and spore germination in *A. flavus* [23,35]. Furthermore, Ren et al. suggested that the suppression of AFB_1_ production by *Trichoderma* results from enzymatic degradation [21]. Notably, the strain *T. citrinoviride* GC-T20 used here was also included in the study by Yue et al., which further supports its AFB_1_-degrading capability [27]. Therefore, the *Trichoderma* strains screened in this work—particularly *T. citrinoviride* GC-T20—are likely to inhibit *A. flavus* and degrade AFB_1_ through a mechanism involving the production of degrading enzymes.

Several studies have investigated the combined application of viable cells (including spores) of *Bacillus* and *Trichoderma*. For instance, Rigobelo et al. reported that co-inoculating soybeans with these microorganisms enhanced plant growth by modifying the soil microbial community [25]. Another microbiological study indicated that while the interaction did not alter plant biomass, it significantly reduced the spore density of pathogenic microbes [36]. Furthermore, Izquierdo et al. examined different combinations of *Bacillus*, *Trichoderma* and their supernatants on Fusarium wilt, revealing that the *Bacillus* supernatant could potentiate the inhibitory activity of *Trichoderma* [37]. However, plate confrontation assays between *Bacillus* and *Trichoderma* strains in our experiments revealed mutual inhibition zones—particularly between *B. subtilis* and *T. citrinoviride*—indicating potential antagonism that could compromise their combined efficacy against *A. flavus*. Furthermore, the culture filtrate of *Trichoderma* strongly inhibited the growth of *Bacillus*, with an inhibition rate exceeding 90%. Conversely, the addition of *Bacillus* culture filtrate to the medium significantly restricted the growth of *Trichoderma*. These results suggest that metabolites present in the culture filtrates of both microorganisms are responsible for their reciprocal inhibition. Thus, we infer that the inhibitory zones observed between *Bacillus* and *Trichoderma* likely arise from certain metabolites produced during cultivation that interfere with each other’s growth and may consequently diminish their individual inhibitory effects on *A. flavus*. Although both *Bacillus* and *Trichoderma* strains effectively inhibit *A. flavus*—with culture filtrates of *B. subtilis* Bs92 and *T. citrinoviride* GC-T20 showing the strongest activity (inhibition rates of 48.61% and 74.03%, respectively)—the observed antagonism between live cells suggests that direct co-application of these two organisms may not be feasible for controlling *A. flavus*.

Therefore, unlike most previous studies that focused on single-species metabolites, we combined the culture filtrates of *B. subtilis* Bs92 and *T. citrinoviride* GC-T20 to enhance the suppression of *A. flavus* growth and AFB_1_ production [31,38]. This approach was designed to circumvent the mutual antagonism observed between the live strains and thereby amplify the overall inhibitory effect on *A. flavus*. Furthermore, although the filtrates of both *B. subtilis* Bs92 and *T. citrinoviride* GC-T20 effectively inhibit the growth of *A. flavus*, their mechanisms for suppressing AFB_1_ production likely differ. Based on the established biocontrol mechanisms of these genera [13,21,27,30], the observed effects suggest that *B. subtilis* Bs92 might inhibit AFB_1_ synthesis potentially through lipopeptides, while *T. citrinoviride* GC-T20 could degrade AFB_1_ via secreted enzymes. This hypothetical framework requires validation through direct chemical analysis of the culture filtrates in future studies. Therefore, when the filtrates are combined, the mixture not only enhances the inhibition of fungal growth, but also integrates the complementary abilities of both strains: the suppression of AFB_1_ synthesis by *B. subtilis* Bs92 and the enzymatic degradation of AFB_1_ by *T. citrinoviride* GC-T20. We acknowledge, however, that testing a broader range of strains is necessary to improve the generalizability of our findings. Future studies will assess the efficacy of this combined microbial formulation against a wider spectrum of aflatoxin-producing fungi and other mycotoxigenic species. In addition, the specific antifungal compounds in the culture filtrates of *B. subtilis* and *T. citrinoviride*—particularly strains Bs92 and GC-T20—remain uncharacterized and require comprehensive identification. We therefore intend to expand the scope of our research, focusing on the isolation and structural elucidation of key antifungal constituents using advanced analytical techniques. In conclusion, the combined application of *Bacillus* and *Trichoderma* culture filtrates provides a promising strategy for inhibiting *A. flavus* and reducing AFB_1_ contamination, offering a potentially effective method for controlling fungal growth and mycotoxin production in food and agricultural products.

## 4. Conclusions

In this study, two strains—*B. subtilis* strain Bs92 and *T. citrinoviride* strain GC-T20—were screened and identified as effective inhibitors of *A. flavus* growth. Based on these findings, we further investigated the interaction between the two strains. Co-culture assays revealed mutual inhibition, whereby metabolites in the culture filtrate of each strain significantly suppressed the growth of the other. Leveraging this antagonistic relationship, we strategically combined culture filtrates from both strains to inhibit both the growth of *A. flavus* and the production of AFB_1_. While the current study focused on establishing the fundamental inhibitory potential of the combined filtrates, we acknowledge that further optimization—such as determining the optimal volumetric ratio of the two filtrates and the effective concentration range against varying pathogen loads—would enhance the practical applicability of this approach. These investigations represent important directions for future research. Although the underlying mechanism remains largely empirical in the absence of metabolomic or transcriptomic data, our results provide a valuable starting point for future mechanistic studies. From an applied perspective, the combined culture filtrates of these two strains hold promise as a bio-fungicide, particularly for the postharvest protection of high-value crops such as maize and peanuts, where they could help reduce aflatoxin contamination at the source.

## 5. Materials and Methods

### 5.1. Materials and Microorganism

The potato dextrose agar (PDA) medium used for solid fungal culture was obtained from Solarbio (Beijing, China). The potato dextrose broth (PDB) medium for liquid fungal culture was sourced from Hopebio (Qingdao, China). The Luria–Bertani (LB) solid medium for culturing *Bacillus* contained 10 g/L peptone, 5 g/L yeast extract, 10 g/L NaCl, and 20 g/L agar, whereas the LB liquid medium was prepared without agar. Peptone and agar were purchased from Maclin (Shanghai, China), yeast extract was acquired from Labgic (Beijing, China), and sodium chloride (NaCl) was supplied by Sinopharm (Beijing, China). AFB_1_ standard with a purity of 99.9% was purchased from Alta Scientific (Tianjin, China). It was dissolved in methanol and stored at −20 °C. All other reagents, including glycerol, methanol, acetonitrile, and acetic acid, were of chromatographic grade and obtained from local suppliers. The *A. flavus* strain was isolated from peanuts. The *Trichoderma* strains employed were those reported by Ren et al. and Yue et al. [21,27]. For preservation, these strains were suspended in 20% (*v*/*v*) glycerol/water solution and preserved at −80 °C in our laboratory in Jinan, China. The *Bacillus* strains used in this study were isolated from the rhizosphere soil of plants, including peanut, corn, and soybean.

### 5.2. Isolation of the Bacillus Strains

According to the method described by Feng et al., 10 g of rhizosphere soil collected from plants (peanut, corn, and soybean) was weighed and suspended in conical flasks containing 90 mL of sterile water [39]. The flasks were shaken at 180 rpm and 36 °C for 15 min, followed by incubation in a 90 °C water bath for 10 min. After cooling to room temperature, the soil suspension was subjected to serial dilution. Aliquots (100 μL) from 10^−1^ to 10^−5^ dilutions were spread on LB agar plates. The LB agar plate was prepared by sterilizing it in a high-pressure autoclave at 121 °C for 30 min and then cooling it. To maximize the recovery of diverse *Bacillus* strains from soil, primary isolation plates were incubated at 29 °C for 24 h, a temperature close to the native soil environment. Subsequently, isolates were cultivated at 37 °C for purification, which is the optimal growth temperature for these strains. Based on colony morphology and size, distinct single colonies were selected and inoculated into LB liquid medium. The axenic cultures were then streaked onto fresh LB agar plates, and after incubation, single colonies were again picked for further purification. This purification process was repeated three times to obtain pure *Bacillus* strains. Finally, the purified strains were mixed with 50% glycerol and stored at −80 °C for long-term preservation.

### 5.3. Molecular Identification of the Bacillus Strains

For the identification of *Bacillus* strains, genomic DNA was first extracted using a Bacterial DNA Extraction Kit (Wuhan, China). PCR amplification was then performed with BGI 2 × Super PCR Mix (Beijing, China) and the bacterial 16S rRNA universal primers 27F (5′-AGAGTTTGATCCTGGCTCAG-3′) and 1492R (5′-TACGGCTACCTTGTTACGACTT-3′). The PCR protocol consisted of initial denaturation at 96 °C for 5 min, followed by 35 cycles of denaturation at 96 °C for 30 s, annealing at 56 °C for 30 s, and extension at 72 °C for 1 min, with a final extension at 72 °C for 5 min. A 3 μL aliquot of each PCR product was analyzed by 1.0% agarose gel electrophoresis to confirm amplification. The products were subsequently purified following a magnetic bead-based protocol and sequenced using primers V4-515F (5′-GTGCCAGCAGCCGCGGTAA-3′) and V4-806R (5′-GGACTACCAGGGTATCTAA-3′). Sequencing services were provided by BGI Genomics (Shenzhen, China). The obtained sequences were compared against the NCBI database using BLAST (version 2.16.1+), and a phylogenetic tree was constructed with MEGA 11.0 software based on homologous sequences [40]. Multiple sequence alignment was performed with CLUSTALW, and the tree was built using the Neighbor-Joining method with 1000 bootstrap replicates; only values exceeding 50% are shown. All *Bacillus* strains used in this study are listed in Table 2.

### 5.4. Inhibitory Effect of the Bacillus Strains on A. flavus

#### 5.4.1. Activation of the Bacillus Strains

The cryopreservation tubes containing 15 strains of Bacillus were retrieved from the −80 °C freezer. Transfer 50 μL of the *Bacillus* culture into a centrifuge tube containing 5 mL of sterile LB liquid medium. Seal the tube and incubate it in a shaker at 37 °C and 180 rpm to activate the bacteria. Subsequently, streak a single colony from each Bacillus strain onto a fresh LB agar plate for isolation. Invert the inoculated plates and incubate them at 37 °C for 24 h.

#### 5.4.2. Activation of the *A. flavus*

The *A. flavus* strain was preserved at −80 °C in 20% glycerol. For revitalization, the stock was thawed and cultured on PDA at 28 °C for 7 days, which is the optimal temperature for *A. flavus* growth and sporulation [20,21]. After abundant sporulation was observed, conidia were harvested by flooding the plate with sterile distilled water and gently scraping the surface. The resulting spore suspension was serially diluted, plated onto PDA, and determined to contain 1 × 10^7^ CFU/mL.

#### 5.4.3. Antagonism of Bacillus spp. Against *A. flavus*

Upon confirmation of well-grown and uncontaminated *Bacillus* colonies, single colonies were picked and transferred into 5 mL of sterile LB liquid medium in 12 mL centrifuge tubes. The tubes were sealed and incubated in a shaker at 37 °C and 180 rpm. The optical density at 600 nm (OD_600_) of the *Bacillus* axenic culture was monitored using a microplate reader (SpectraMax M2, San Jose, CA, USA), and incubation was continued until it reached approximately 0.8. The experiment on the antagonism of *Bacillus* spp. against *A. flavus* was conducted based on the method of Ren et al. for *Trichoderma* spp., with modifications [21]. Small circular filter paper discs (5 mm in diameter) were punched from sterile filter paper using a hole puncher, placed in a glass Petri dish, and sterilized by autoclaving at 121 °C for 15 min. Four discs were symmetrically positioned on the PDA plate, each 25 mm from the center, to ensure even distribution. Then, 10 μL of *Bacillus* axenic culture was applied to each disc, ensuring full coverage without overflow or aggregation. Subsequently, 10 μL of the *A. flavus* spore suspension was inoculated at the center of each PDA plate. In the control group, only the *A. flavus* spore suspension was inoculated without any *Bacillus*. All plates were incubated at 28 °C in a constant-temperature incubator. After 7 days, the diameter of the *A. flavus* colonies was measured. The inhibition rate (IR) of *Bacillus* strains against *A. flavus* was calculated using the following formula:

IR (%) = [(R_1_ − R_2_)/R_1_] × 100

where R_1_ represents the colony diameter in the control group (without *Bacillus*), and R_2_ represents the colony diameter in the treatment group (with *Bacillus*).

### 5.5. Inhibitory Effect of the Bacillus Strain Culture Filtrates on A. flavus

The method for preparing culture filtrates was adapted from Sawai et al. [34]. The *Bacillus* axenic culture (OD_600_ ≈ 0.8) was then transferred into 200 mL of sterile LB liquid medium for scale-up fermentation. After incubation under the same conditions (37 °C, 180 rpm) for 3 days, a broth rich in metabolites was obtained. To remove mycelial debris and large particles, the axenic culture was first filtered through sterile qualitative filter paper. The filtrate was then centrifuged at 8421× *g* for 30 min, and the resulting supernatant was collected for subsequent experiments. After the sterilized PDA medium had cooled to approximately 60 °C, 15 mL of the liquefied PDA was thoroughly mixed with 5 mL of the *Bacillus* culture filtrate and promptly poured into a Petri dish. Once solidified, an activated mycelial plug of *A. flavus* was placed at the center of the plate. A control group was prepared using PDA plates containing only 20 mL of PDA medium without any *Bacillus* culture filtrate. All plates were sealed and incubated in the dark at 28 °C for 7 days. After incubation, the growth of *A. flavus* was assessed, and the colony diameter was measured. The inhibition rate (IR) of the *Bacillus* culture filtrate against *A. flavus* was calculated as follows:

IR (%) = [(R_1_ − R_2_)/R_1_] × 100

where R_1_ represents the colony diameter in the control group (PDA medium only), R_2_ represents the colony diameter in the treatment group (PDA medium supplemented with *Bacillus* culture filtrate).

### 5.6. Inhibitory Effect of the Trichoderma Strain Culture Filtrates on A. flavus

Prior to evaluating the inhibitory effect of *Trichoderma* culture filtrates on *A. flavus*, the preserved *Trichoderma* strains were first activated. Each of the 15 preserved *Trichoderma* strains was inoculated onto individual PDA plates. The inoculated plates were sealed with parafilm to prevent contamination and incubated at 28 °C for 5 days to activate the strains for subsequent experiments. A method similar to that used for *Bacillus* was employed to prepare the *Trichoderma* culture filtrate. Using a 0.5 cm diameter hole puncher, 10 mycelial plugs were taken from the actively growing edge of each *Trichoderma* colony and transferred into a 250 mL conical flask containing 100 mL of sterile PDB medium. The flasks were incubated in a shaker at 28 °C and 180 rpm for 7 days. After cultivation, the mycelia were removed by filtration through sterilized filter paper, and the resulting culture broth was further filtered through a 0.22 μm microporous membrane to obtain a cell-free culture filtrate, which was stored for subsequent experiments. For the inhibition assay, 15 mL of sterilized liquefied PDA medium (cooled to approximately 60 °C) was thoroughly mixed with 5 mL of *Trichoderma* culture filtrate and poured into a Petri dish. Once solidified, 10 μL of *A. flavus* spore suspension was inoculated at the center of the plate. The control group consisted of PDA plates containing only 20 mL of PDA medium without any *Trichoderma* culture filtrate. All plates were sealed and incubated at 28 °C for 7 days, after which the colony diameter of *A. flavus* was measured. The inhibition rate (IR) of the *Trichoderma* strain culture filtrate against *A. flavus* was calculated using the following formula:

IR (%) = [(R_1_ − R_2_)/R_1_] × 100

where R_1_ represents the colony diameter in the control group (PDA medium only), and R_2_ represents the colony diameter in the treatment group (PDA medium supplemented with *Trichoderma* culture filtrate).

### 5.7. Interactions Between Bacillus spp. and Trichoderma spp.

#### 5.7.1. Dual Culture Assay between *Bacillus* Strains and *Trichoderma* Strains

Based on the above experiments, two *Bacillus* strains (Bs92 and BvL1) and five *Trichoderma* strains (GC-T20, GC-T19, GC-T21, GC-T2, GC-T4), which exhibited the strongest inhibitory effects against *A. flavus*, were selected for further interaction assays. The experimental setup was adapted from the previously described antagonism assay between *Bacillus* and *A. flavus*. On each PDA plate, 10 μL of activated *Bacillus* axenic culture was applied onto each of four sterile filter paper discs (previously positioned 25 mm from the center). An activated mycelial plug of *Trichoderma* (6 mm in diameter) was then placed at the center of the same plate. In the control group, only the *Trichoderma* mycelial plug was inoculated on the PDA medium. All plates were incubated at 28 °C for 7 days. Photographs were taken on days 3 and 7 to assess the presence of any inhibition zones between the *Bacillus* and *Trichoderma* strains.

#### 5.7.2. Inhibitory Activity of *Trichoderma* Strains’ Culture Filtrates Against *Bacillus* Strains

Meanwhile, four strains exhibiting strongest inhibitory activity against *A. flavus* were selected—two *Bacillus* strains (Bs92, BvL1) and two *Trichoderma* strains (GC-T20, GC-T19). To perform the liquid confrontation assay between *Bacillus* and *Trichoderma*, the *Bacillus* axenic culture and *Trichoderma* culture filtrate were prepared in advance. A mixture was prepared by combining 30 mL of sterile LB liquid medium with 10 mL of *Trichoderma* culture filtrate. Then, 1 mL of *Bacillus* axenic culture was inoculated into the mixed medium. In the control group, 1 mL of *Bacillus* axenic culture was added to 40 mL of sterile LB liquid medium without any fungal filtrate. All liquid cultures were incubated in a shaker at 180 rpm and 37 °C for 7 days. After incubation, the OD_600_ of each culture was measured to estimate bacterial concentration. Subsequently, the cultures were serially diluted (10^−1^ to 10^−6^). A 100-μL aliquot of the 10^−6^ dilution was evenly spread onto LB agar plates, which were then incubated at 37 °C for 24 h. The number of *Bacillus* colonies was then counted.

#### 5.7.3. Inhibitory Activity of *Bacillus* Strains’ Culture Filtrates Against *Trichoderma* Strains

Following the method described in Section 2.5, activated mycelial plugs of *Trichoderma* strains were placed at the center of plates containing a mixed medium of PDA and *Bacillus* culture filtrate. The control group contained only PDA medium without any bacterial filtrate. After incubation at 28 °C for 3 and 7 days, images were taken for documentation, and the inhibition rate of *Bacillus* strains against *Trichoderma* strains was calculated using a formula analogous to that described previously.

### 5.8. Inhibitory Activity of Bacillus and Trichoderma Strains’ Culture Filtrates Against A. flavus

Culture filtrates were prepared from the *Bacillus* strain Bs92 and *Trichoderma* strain GC-T20, which showed the strongest inhibitory activity against *A. flavus*. A mixed culture filtrate was prepared by combining equal volumes of each filtrate. As described previously (Section 2.5), 15 mL of molten (uncooled) PDA was thoroughly mixed with 5 mL of each of the three culture filtrates (*Bacillus*, *Trichoderma*, and mixed) and immediately poured into Petri dishes. After solidification, an activated mycelial plug of *A. flavus* was inoculated on 20 mL of PDA medium without any culture filtrate. All plates were sealed and incubated in the dark at 28 °C for 7 days. The inhibition rate (IR) of each culture filtrate on the radial growth of *A. flavus* was calculated as follows:

IR (%) = [(R_1_ − R_2_)/R_1_] × 100

where R_1_ represents the colony diameter in the control group (PDA only), R_2_ represents the colony diameter in the treatment group (PDA supplemented with a culture filtrate).

For the liquid culture assay, 10 mL of each filtrate (*Bacillus*, *Trichoderma*, and mixed) was blended with 30 mL of sterile LB liquid medium. A control containing only 40 mL of LB medium was also prepared. Then, 20 μL of *A. flavus* spore suspension was added to each flask, and cultures were incubated in a shaker at 180 rpm and 28 °C for 7 days. After incubation, the AFB_1_ concentration in the filtered culture broth was quantified using high-performance liquid chromatography (HPLC). The inhibition rate of AFB_1_ production was calculated using the formula:

IR (%) = [(R_1_ − R_2_)/R_1_] × 100,

where R_1_ and R_2_ represent the AFB_1_ concentration in the control and treatment groups, respectively.

### 5.9. Detection of AFB_1_ Concentration by HPLC

The concentration of AFB_1_ in the liquid culture medium was determined according to the method of Yue et al. [27]. Briefly, after cultivation, the medium was filtered through filter paper to remove the mycelia of *A. flavus*. The filtrate was then diluted tenfold with a mixed reagent (acetonitrile–water–acetic acid, 44.5:54.5:1, *v*/*v*/*v*) and passed through a 0.22 μm filter prior to injection. AFB_1_ was quantified using high-performance liquid chromatography (HPLC) (Agilent Technologies, Santa Clara, CA, USA). The fluorescence detector was set at excitation and emission wavelengths of 360 nm and 440 nm, respectively. Separation was performed on a C-18 column (4.6 mm inner diameter × 250 mm length, 5 μm particle size). The injection volume was 3 μL, and the mobile phase consisted of methanol and water (45:55, *v*/*v*) delivered at a flow rate of 0.3 mL/min over a 10-min run time. Quantification was based on the peak area at the retention time of the AFB_1_ standard using an external calibration curve. Under the applied conditions, the AFB_1_ standard eluted at 3.7 min. Sample concentrations were determined by comparing peak areas at this retention time against a five-point calibration curve (0.2–10.0 ng/mL), which showed excellent linearity. The limit of quantification (LOQ) was 0.2 ng/mL, defined as a signal-to-noise ratio of 10:1.

### 5.10. Statistical Analysis

All experiments were conducted in triplicate, with data expressed as mean ± standard deviation (SD). Statistical analysis was performed using IBM SPSS Statistics 26 (SPSS Inc., Chicago, IL, USA), and graphs were generated with Origin Pro 2019b (Origin Lab, Northampton, MA, USA). Differences among groups were assessed by analysis of variance (ANOVA) followed by the Tukey–Kramer post hoc test, with statistical significance defined as *p* < 0.05.

## Figures and Tables

**Table 1:** Impact of different *Trichoderma* culture filtrates on the OD_600_ value of *Bacillus*.

Treatment	Against BvL1	Inhibition Rates (%)	Against Bs92	Inhibition Rates (%)
CK	1.18 ± 0.02	\	1.19 ± 0.02	\
GC-T19	0.08 ± 0.00	93.22 ± 0.01	0.07 ± 0.00	93.78 ± 0.00
GC-T20	0.07 ± 0.00	93.51 ± 0.00	0.07 ± 0.01	94.03 ± 0.01

Data are presented as mean ± SD (*n* = 3).

**Table 2:** Strains of *Bacillus* used in this study.

Species	Strain	Source	Number
*Bacillus subtilis*	JF8-3	Rhizosphere soil of corn	PX588886
	JF7	Rhizosphere soil of peanuts	PX588885
	JF9-1	Rhizosphere soil of peanuts	PX588887
	Bs92	Rhizosphere soil of soybeans	PX588888
*Bacillus velezensis*	BvL1	Rhizosphere soil of peanuts	PX588889

## Data Availability

The original contributions presented in this study are included in the article. Further inquiries can be directed to the corresponding author.
